# Drosophila *chem* mutations disrupt epithelial polarity in Drosophila embryos

**DOI:** 10.7717/peerj.2731

**Published:** 2016-12-01

**Authors:** José M. Zamudio-Arroyo, Juan R. Riesgo-Escovar

**Affiliations:** Developmental Neurobiology and Neurophysiology, Instituto de Neurobiología, Universidad Nacional Autónoma de México, Querétaro, Querétaro, México

**Keywords:** Cell polarity, Epithelial cell, Dorsal closure, Head involution

## Abstract

Drosophila embryogenesis has proven to be an extremely powerful system for developmental gene discovery and characterization. We isolated five new EMS-induced alleles that do not complement the *l(3R)5G83* lethal line isolated in the Nüsslein-Volhard and Wieschaus screens. We have named this locus *chem*. Lethality of the new alleles as homozygous zygotic mutants is not completely penetrant, and they have an extended phenocritical period. Like the original allele, a fraction of mutant embryos die with cuticular defects, notably head involution and dorsal closure defects. Embryonic defects are much more extreme in germline clones, where the majority of mutant embryos die during embryogenesis and do not form cuticle, implying a strong *chem* maternal contribution. *chem* mutations genetically interact with mutations in cytoskeletal genes (*arm*) and with mutations in the epithelial polarity genes *coracle, crumbs,* and *yurt*. *chem* mutants dorsal open defects are similar to those present in *yurt* mutants, and, likewise, they have epithelial polarity defects. *chem^1^* and *chem^3^* mutations suppress *yurt^3^*, and *chem^3^* mutants suppress *crumbs^1^* mutations. In contrast, *chem^1^* and *coracle^2^* mutations enhance each other. Compared to controls, in *chem* mutants in embryonic lateral epithelia Crumbs expression is mislocalized and reduced, Coracle is increased and mislocalized basally at embryonic stages 13–14, then reduced at stage 16. Arm expression has a similar pattern but levels are reduced.

## Introduction

Embryonic development was systematically explored for the first time in Drosophila over thirty years ago in the Nüsslein-Volhard and Wieschaus genetic screen (Jürgens et al., 1984; Nusslein-Volhard & Wieschaus, 1980; Nüsslein-Volhard, Wieschaus & Kluding, 1984; Wieschaus, Nüsslein-Volhard & Jürgens, 1984). Most genes relevant for embryonic development were originally isolated then, and subsequent work have characterized many of them. Besides originally categorizing mutant phenotypes and gene classes, these studies lead to many mechanistic insights and principles of developmental processes.

Some key findings illustrated by the mutations isolated are the importance of epithelia, epithelial polarization and movement, and changes in cell shape (Knust, 2003). Epithelial cells that undergo concerted movements and changes in shape become polarized first (Müller, 2003). Epithelial polarization establishes two domains: apical and basolateral (Le Bivic, 2005; Müller & Bossinger, 2003). These are generally recognized and assessed by the presence of marker proteins (Muller & Wieschaus, 1996).

Embryos with strongly disrupted apico-basal polarity do not develop, and result in lethal mutant phenotypes where only small pieces of cuticle are synthesized. Genetic analysis has uncovered that genes necessary for epithelial polarity code for cytoskeletal proteins and their regulators, like the par-3 protein Bazooka (Kuchinke, Grawe & Knust, 1998) or the EGF and laminin domains-containing protein Crumbs (Tepass, Theres & Knust, 1990). Mutations in genes with less extreme phenotypes may result in impaired cell movement and cell shape changes, hampering or preventing embryonic dorsal closure and head involution (Rios-Barrera & Riesgo-Escovar, 2013). Signaling genes necessary for orchestrating these processes (like the JNK pathway (Rios-Barrera & Riesgo-Escovar, 2013)) regulate cytoskeletal proteins, like the FERM-domain proteins Coracle (Fehon, Dawson & Artavanis-Tsakonas, 1994) and Yurt (Hoover & Bryant, 2002).

One of the lethal lines isolated from the Nüsslein-Volhard/Wieschaus screens and not characterized further is *l(3R)5G83* (Jürgens et al., 1984). They isolated only one allele with dorsal closure defects. We isolated five new mutant alleles with embryonic phenotypes. A fraction of these mutant embryos have lethal head involution or dorsal open phenotypes, and have an extended phenocritical period. Germline clones derived from the mutant alleles have much stronger embryonic lethal phenotypes. In addition, we show that these alleles genetically interact with epithelial polarity genes, and have epithelial polarity defects.

## Materials & Methods

### Genetics, genetic screen, and strains

The *l(3R)5G83* mutant allele was obtained from the Tübingen stock center. We mutagenized *y*, *w* control stock males with 25 mM ethyl methane sulphonate (EMS) according to Lewis & Bacher (1968). We crossed mutagenized males to third chromosome balancers, and F1 males over the balancer were crossed individually to *y*, *w*; *l(3R)5G83*, FRT82/TM3, *Sb*^1^, *Ser*^1^ females, and progeny scored for lack of complementation. We named the locus *chem*. All mutant *chem* alleles were recombined onto FRT82 chromosomes. In so doing, we also “cleaned up” the mutagenized chromosomes from putative second side mutations, as the FRT82 containing chromosome we used for recombination renders flies viable and fertile as homozygotes. All third chromosomes stocks used were balanced over a “green” third chromosome balancer (one expressing GPF embryonically; Bloomington stock # 6663) before being used in experiments. To score lethality egg lays were performed, and dead and surviving mutant embryos (scored by the absence of GFP) were counted. For postembryonic lethality first instar mutant larvae were transferred to food vials (less than 30 larvae per vial), or to fresh egg-laying plates with yeast, and cultured at 25 °C, 50% humidity, 12:12 light:dark cycle conditions. Dead and surviving organisms were scored.

We obtained *yurt* ^3^ from the Tübingen stock center, and *coracle*^2^ (*cora*) from R. Fehon. We obtained *crumbs*^8*F*105^ from the Bloomington Drosophila stock center (stock #7099), on a marked *rucuca* third chromosome without *ca*. We recombined *chem*^1^ with *crumbs*^8*F*105^ for genetic interaction studies. We independently recombined *yurt*^3^ with *chem*^1^ and *chem*^3^ for genetic interaction studies, and double-balanced *cora*^2^, *chem*^1^ and *chem*^3^ using a “green” double balancer (stock #5703 from Bloomington). For genetic interaction studies, we crossed the “green” balanced double heterozygote stocks to similarly “green” balanced single heterozygotes independently, and the double heterozygote with itself separately. All flies were cultured in freshly yeasted yeast-molasses standard food medium at room temperature (22–25 °C), 50% humidity, and in a 12:12 hrs light:dark cycle.

### Cuticular analysis

We recovered egg lays of the different stocks and crosses, and selected non-GFP fluorescent embryos. We then prepared embryonic cuticles from dead embryos as in Riesgo-Escovar et al. (1996), except that we used PVA as mounting medium (BioQuip). The slides were viewed using dark field microscopy and photographed.

### Germline clones

We recovered *chem*^1^, *chem*^3^ and *chem*^5^ germline clones with and without paternal rescue by crossing to an FRT82 *ovo*^*D*1^ stock as in Chou, Noll & Perrimon (1993). We generated germline clones homozygous for *chem*^1^, *chem*^3^ and *chem*^5^, and also heteroallelic crosses with *chem*^1^ and *chem*^3^ mutant germline derived oocytes crossed to *chem*^3^ and *chem*^5^ paternally.

### Immunohistochemistry and microscopy

**Figure 1:**
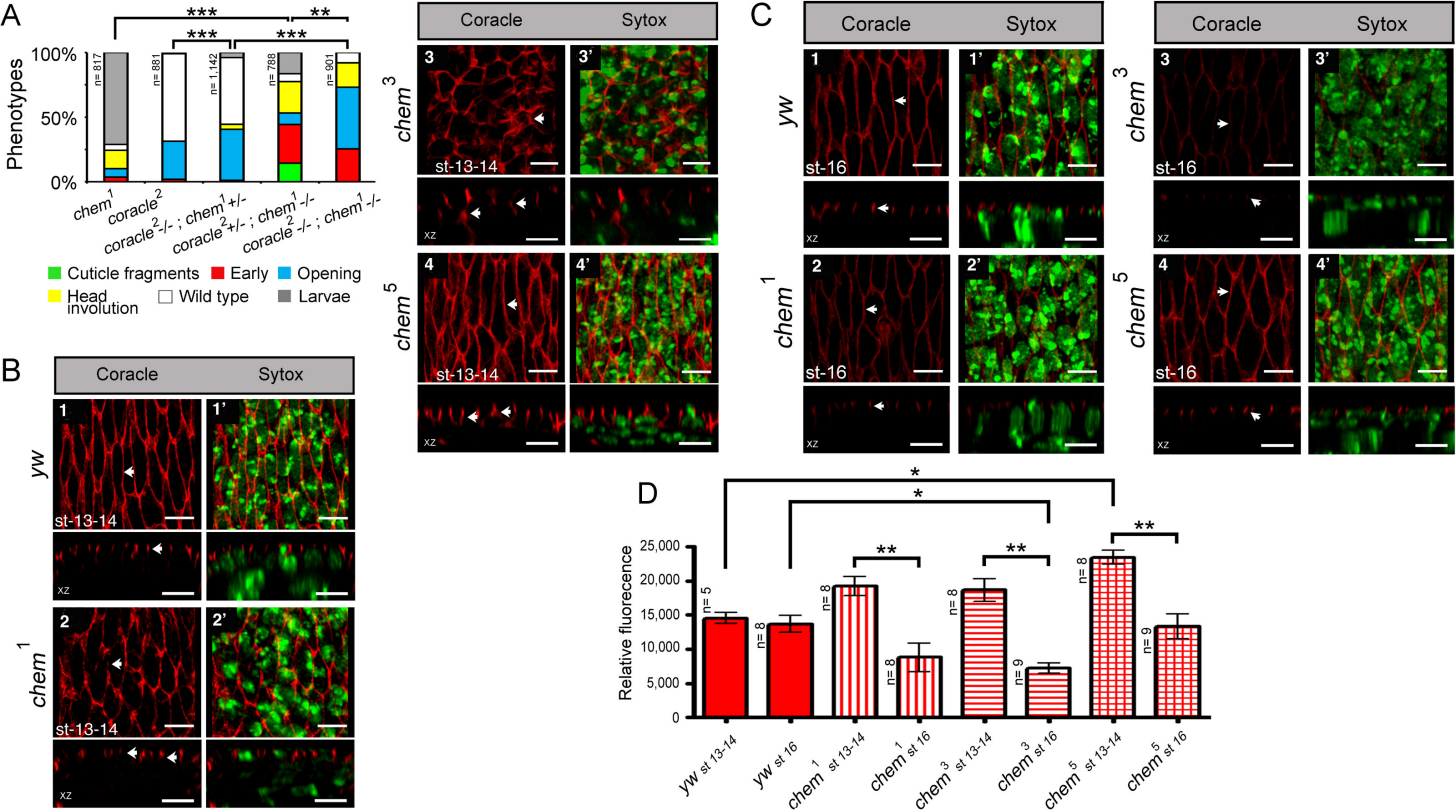
*chem* and *cora* enhance the mutant phenotypes of each other. (A) Both *cora^2^* and *chem*^1^ as heterozygotes significantly enhance homozygous mutant conditions of the other allele as embryos. The double homozygote has an intermediate phenotype between *chem*^1^and *cora^2^*. n is written to the left of each genotype column. (B) In *chem* mutants of stages 13–14 of embryogenesis, the lateral epithelial architecture is disrupted (compare control panel *y w*, to *chem*^1^, *chem*^3^ and *chem*^5^ panels). For each genotype the top views (1–4) are projections of confocal stacks showing Cora staining in a head on view, with the left panels (1^′^–4^′^) also showing a nuclear Sytox-Green staining. Bottom panels show XZ projections of the stacks, to localize the Cora and Cora together with nuclei (Sytox) channels taken from the same stacks as above. Representative examples are shown, and an n of 8 embryos per genotype was imaged. The white arrows show details of the Cora staining. Notice basally mislocalized Cora staining in *chem*^3^ and *chem*^5^. Scale bar is 5 micrometers throughout. (C) In *chem* mutants of stage 16 of embryogenesis stained with Cora antibodies, there is a dramatic reduction of Cora expression. As in (B), for each genotype the top views (1–4) are projections of confocal stacks showing Cora staining in a head on view, with the left panels (1^′^–4^′^) also showing a nuclear Sytox-Green staining. Bottom panels show XZ projections of the stacks, to localize the Cora and Cora together with nuclei (Sytox) channels taken from the same stacks as above. Representative examples are shown, and an n of 8–9 embryos per genotype was imaged. The white arrows show details of the Cora staining. Scale bar is 5 micrometers throughout. (D) Quantification of Cora staining of experiments in (B and C). Notice significant reduction of Cora staining in all *chem* mutant embryos as stage 16 compared to stages 13–14. n is shown to the left of each column.

**Figure 2:**
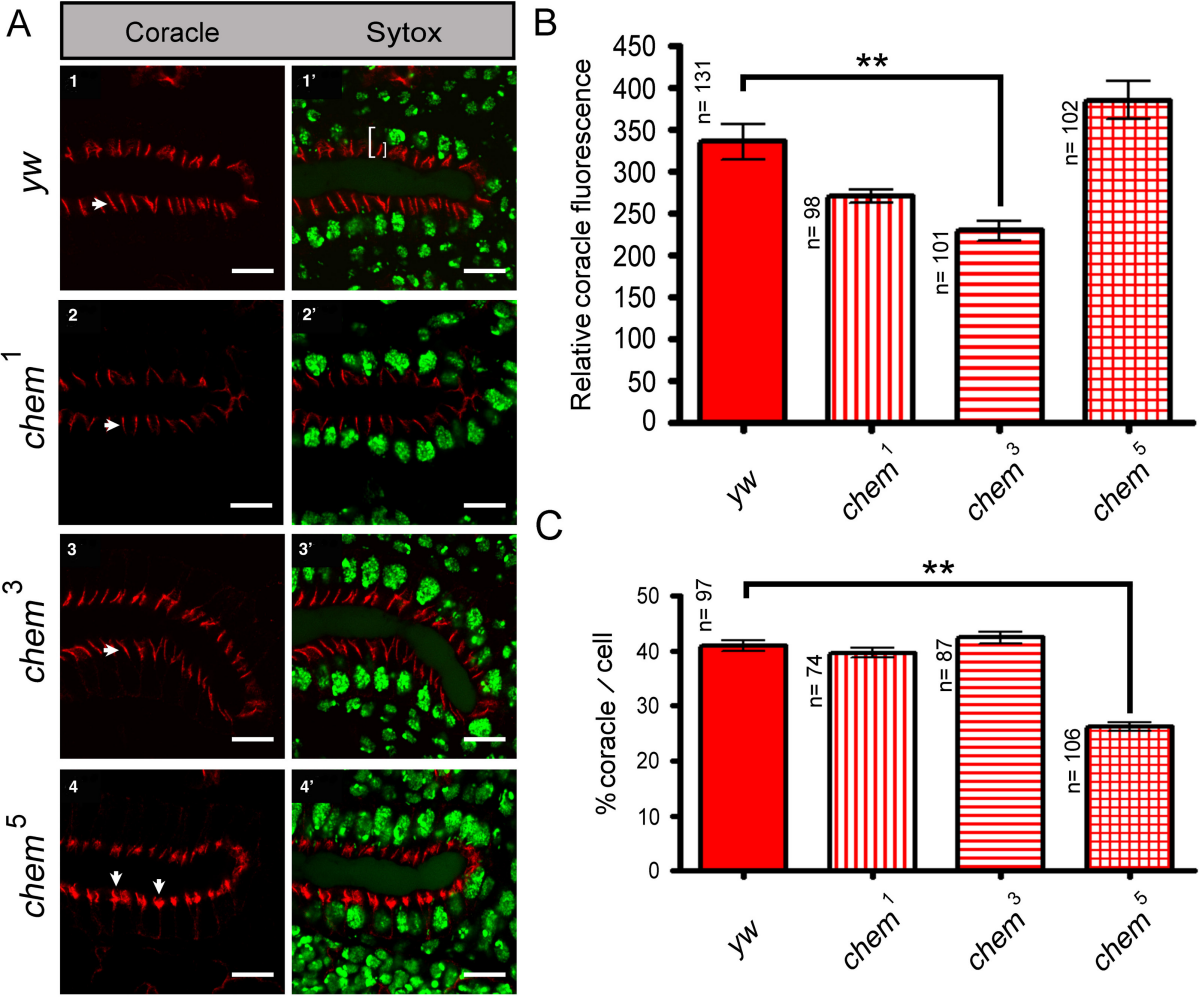
Cora staining in stage 16 salivary glands. (A) 1–4 show confocal optical sections for the Cora channel (red); arrows point to Cora staining in all panels. 1^′^–4^′^ show the corresponding optical section with the Sytox (nuclei) channel added. In 1^′^ the left white bracket illustrates the length measurement used to calculate the “partial length of cell” measurement, from the apical, lumenal site of Cora staining to the base of the salivary glands nuclei underneath. The smaller white right bracket in the same panel illustrates the Cora staining measurements used for Fig. 2C, showing the extension of the Cora staining in the cells. Scale bar is 10 micrometers. (B) Shows the quantitation of the relative Coracle staining using an area of 12.53 square microns for each measurement from optical sections as the ones illustrated in panels 1–4. The reduction of staining in *chem*^3^ is significant. n is written to the left of each genotype column. (C) The extension of Cora staining in relation to the cell length. In order to control for cell size differences, we measured in optical sections like the ones depicted in panels 1^′^–4^′^ the length of the cell from the apical, lumenal side of the Cora staining to the base of the underlying salivary gland nuclei, and we compared these measurements to the extension of the Cora staining. We graphed the percentage of the “partial cell length” thus measured to the Cora staining extension. *chem*^5^ extension is significantly reduced compared to the control. n is written to the left of each genotype column.

Embryos were fixed in two different ways. For staining with anti-Crumbs (Cq4) and anti-Armadillo (N27A1) antibodies (both from the Developmental Studies Hybridoma Bank), we fixed the dechorionated embryos by a two second heat treatment at 93.4 °C, basically as described in Miller, Field & Alberts (1989). For the anti-Coracle (C566.9) and anti-Fasciclin 3 (7G10) stainings (antibodies also from the Developmental Studies Hybridoma Bank), we fixed dechorionated embryos according to Krahn et al. (2010). The rest of the protocol was according to Miller, Field & Alberts (1989). Anti-Crumbs was used at a 1:20 dilution, anti-Coracle at a1:200, anti-Fasciclin 3 at 1:200, and anti-Armadillo at a1:20. Secondary antibody was anti-mouse conjugated with Cy-3 (Invitrogen) at 1:1,000. For nuclei, we used Sytox-Green (1:3,000) according to the manufacturer’s instructions (Invitrogen). Embryos were mounted in Vectashield (Vector Laboratories), and imaged using a Zeiss 780 confocal microscope with 25× and 63× objectives. Images were acquired with Zeiss software, and manipulated using ImageJ (NIH). We used stage 13–14 embryos for the Crumbs, Coracle, and Armadillo antibody stainings; for the Fasciclin 3 staining, we used stages 15–16 embryos, except for Fig. S1, where stages 13–14 embryos were used. Finally, in Figs. 1C and 2 stage 16 embryos were used.

### Fluorescence intensity measurements

For fluorescence intensity measurements in the lateral epithelium of stages 13–14 embryos stained with antibodies against Armadillo, Coracle and Crumbs and stage 16 embryos stained with antibodies against Coracle, we generated 10 micrometers thick stacks of optical sections. Using Zeiss software a maximal intensity projection was generated. Then, 25 square micrometer areas were used with the set measurement parameters of ImageJ to calculate a fluorescence intensity value. For amnioserosa fluorescence intensity values from stages 13–14 embryos, 4-micrometer thick stacks were used. Using Zeiss software as above, we generated maximal projections of the stacks, and a 20.03 × 64.78 micrometer area was selected that only had amnioserosa cells for the fluorescense intensity measurements as described for the lateral epithelium. The intensity of Cora staining in stage 16 salivary glands was measured using single optical sections that basically bisected the gland, in all cases using an area of 12.53 square microns. To control for differences in staining, the staining was repeated several times, and data taken from appropriately staged embryos from the different experiments.

### Cell length measurements

We used Fas 3 stained lateral epithelial cells of stages 13–14 embryos for cell length measurements. Z projections were generated from 12 micrometer thick stacks. We divided the stack into four equivalent sections, and made orthogonal views form each section, such that we had from every stack three orthogonal sections. Since they comprised different cells from the lateral epithelium, we used them to measure the Fas 3 staining length, tracing a line through the middle of the staining from top to bottom following the Fas 3 staining. Fas 3 does not stain the basal membrane of the cells, neither does it stain the apical membrane, so the measurement is a partial measure of the actual cell length. We used these to assess cell length. We also used a different measurement for assessing partial cell length in salivary gland epithelia: We measured the distance from the base of the epithelial nuclei to the end of the Cora expression at the luminal side of the epithelial cells, and compared that to the extension of the Cora expression in the same cells. We then calculated what percentage of this partial cell length had Cora expression.

### Statistics

Statistical analysis of differences between distributions (germline clones with and without paternal rescue, and genetic interaction experiments) was done with the SAS implemented chi-square procedure (the distributions being non-normal), with significance set at *P* < 0.05. Least square means plus or minus error of the mean was used to analyze differences between genotypes. Fluorescence differences were analyzed using one-way ANOVA with a Tukey post-hoc test (SAS Online Doc. 9.1.3; SAS Institute, Cary, NC, USA).

## Results

### *chem* alleles

**Figure 3:**
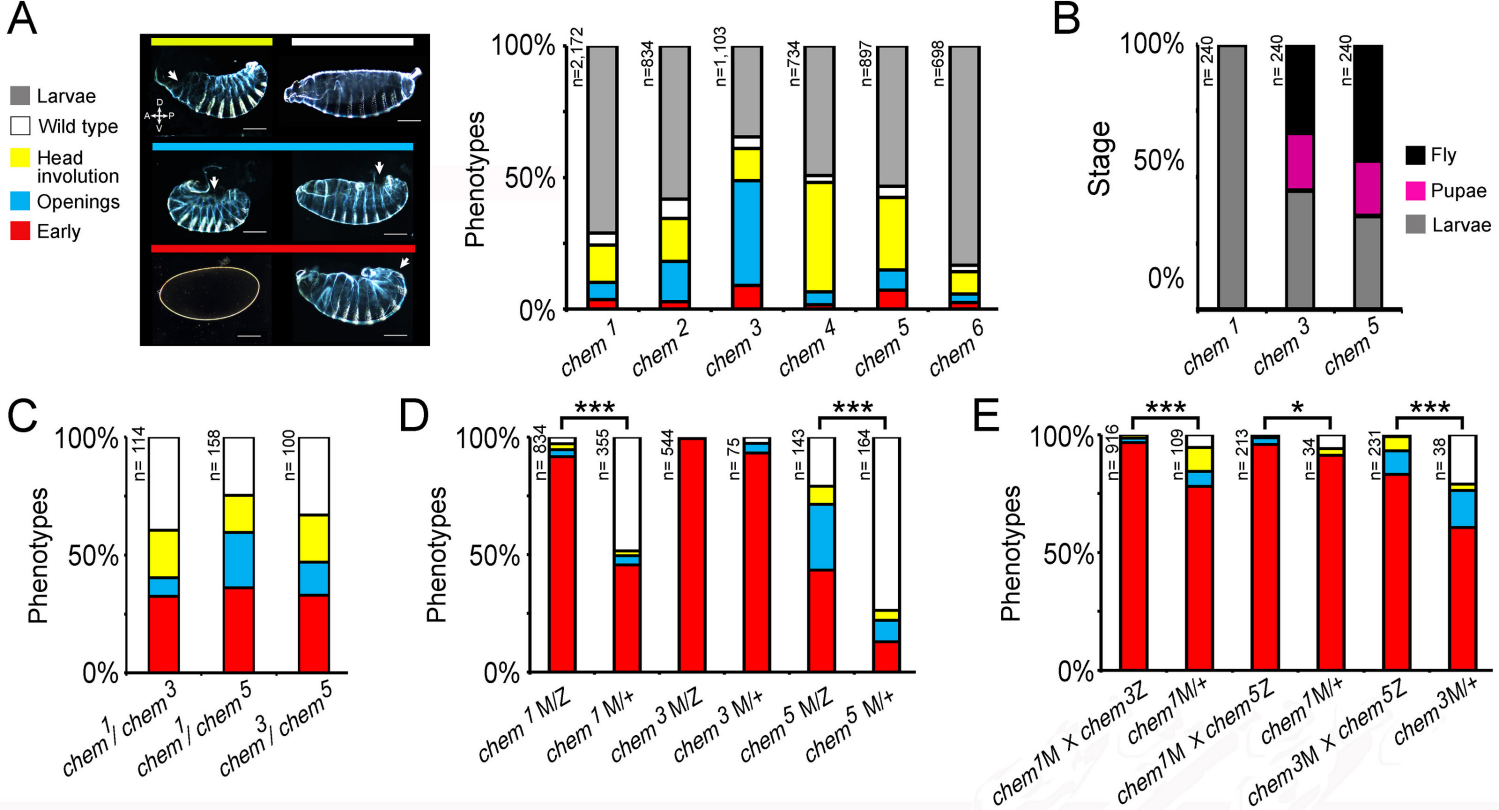
A genetic screen yields new *chem* alleles. (A) Shows the percentage of embryonic lethal phenotypes of six different *chem* mutant alleles, and the percentage of surviving mutant first instar larvae. n for each genotype is marked to the left of each column. The different cuticular phenotypes are illustrated in the left hand side panel. Anterior is to the left, and dorsal is up. Most mutant embryos that die with cuticular phenotypes have dorsal holes (either dorsal open or head involution defective). Scale bar is 100 micrometers. (B) Homozygous mutant embryos were selected from balancer-containing siblings by virtue of lack of GFP expression (the balancers used in these experiments all had embryonic GFP expression). Surviving first instar mutant larvae were selected from egg lays, and cultured in fresh food vials, and scored for death of larvae and pupae, and eclosing adults. *n* = 240 larvae per genotype. For (C–E) color code is the same as in (A), and n is written to the left of the corresponding column. (C) *chem* heteroallelic combinations show the same embryonic phenotypes as *chem* homozygous mutants. The graph depicts dead embryos phenotypes. Surviving larvae die before reaching the adult stage. In (D–E) ‘M’ refers to maternally supplied *chem* germline clone allele; ‘Z’ refers to paternally provided mutant *chem* allele, and ‘+’ refers to a wild type paternally supplied *chem* allele (paternal rescue). (D) *chem* germline clones have very early embryonic phenotypes, much stronger than corresponding homozygous zygotic mutants. Notice that in *chem*^3^ germline clones all embryos have early lethal phenotypes. Paternal rescue with a wild type *chem* allele has a significant effect (reduction of severity of mutant phenotypes) for *chem*^1^ and *chem*^5^. (E) Heteroallelic mutant combinations of mutant germline clones and paternally provided *chem* alleles, with and without paternal rescue, show similar embryonic early phenotypes as homozygous mutants. Paternal rescue is significant for all combinations tested. In (C) and (E) we studied the *chem*^1^ mutant allele in heteroallelic combination with *chem*^3^ and *chem*^5^, which have different genetic backgrounds, making very highly improbable that a putative common second site mutation would be responsible for the observed phenotypes. For all figures, significance is as follows: * *p* ≤ 0.05, ** *p* ≤ 0.001, *** *p* ≤ 0.0001.

We recombined the original *l(3R)5G83* allele (now named *chem*^1^) onto an FRT82 chromosome and found that mutant cuticular phenotypes were present in about a quarter of mutant embryos. Homozygous *chem*^1^ is lethal, with an extended phenocritical period encompassing embryos and larvae (Figs. 3A and 3B). We used this allele to isolate five new alleles of the *chem* (*l(3R)5G83*) complementation group in an EMS mutagenesis (Table S1). All new alleles fail to complement *chem*^1^, and complementation crosses between them only yield 0–5.2% surviving transheterozygotes of an expected 33.3% (Table S1).

All five new alleles have similar embryonic phenotypes (Fig. 3A). Most zygotic mutant embryos that die with cuticular phenotypes have dorsal open and head involution defects. Since for all alleles the cuticular phenotypes look like a small boat, we named the locus *chem. chem* means ‘small canoe’ in the Mayan language. Thus, we named *l(3R)5G83* as *chem*^1^, and the five new alleles as *chem*^2−6^ (Fig. 3A).

We also studied surviving mutant *chem*^1^, *chem*^3^ and *chem*^5^ larvae. We cultured these surviving mutant larvae in isolation, without balancer-containing siblings (Fig. 3B). *chem*^1^ larvae all die during the larval period, whereas for *chem*^3^ and *chem*^5^ mutant larvae a fraction (around 40%) dies as larvae, a fraction (around 20%) dies as pupae, and a fraction reaches adulthood (Fig. 3B), and dies after a few days. When the mutant larvae of these alleles are co-cultured with balancer-containing siblings, they do not survive.

We then analyzed heteroallelic mutant combinations, examining embryonic mutant phenotypes for *chem*^1^/*chem*^3^, *chem*^1^/*chem*^5^, and *chem*^3^/*chem*^5^(we focused on embryonic mutant phenotypes; a fraction of heteroallelic embryos survived embryogenesis and died mostly as larvae, as do *chem* homozygous mutants). These heteroallelic combinations show similar embryonic mutant phenotypes as mutant homozygotes (Fig. 3C), except that early mutant phenotypes are more common. We conclude that heteroallelic *chem* mutants do not complement and present the same mutant phenotypes as homozygotes.

The embryonic mutant phenotypes we see are not completely penetrant, and a small fraction of mutant individuals reaches adulthood and dies a few days later. We wondered whether there is a *chem* maternal contribution that obscures early *chem* requirements. To test this, we generated germline clones for *chem*^1^, *chem*^3^, and *chem*^5^, with and without paternal rescue (Fig. 3D). The embryonic mutant phenotypes are strikingly stronger compared to corresponding zygotic mutants, with ‘early’ phenotypes (no cuticle formed, mostly) in almost all embryos, or very prevalent (specifically, over 90% in *chem*^1^ and *chem*^3^, and close to 50% in *chem*^5^). Wild type paternal rescue had a strong effect, significantly lessening the mutant phenotypes for *chem*^1^ and *chem*^5^. From this, we can construct an allelic series as follows: *chem*^3^ > *chem*^1^ > *chem*^5^. For zygotic phenotypes (Fig. 3A), the allelic series is as follows: *chem*^3^ > *chem*^4^ > *chem*^5^ > *chem*^2^ > *chem*^1^ > *chem*^6^. This places *chem*^3^ as the strongest allele of the series.

We also studied germline clones using the same heteroallelic combinations as in Fig. 3C (Fig. 3E). Again, mutant phenotypes are stronger than the corresponding zygotic mutant combinations. Again, as well, paternal rescue had a significant effect lessening the extent of mutant phenotypes.

### *chem* and *yurt*

**Figure 4:**
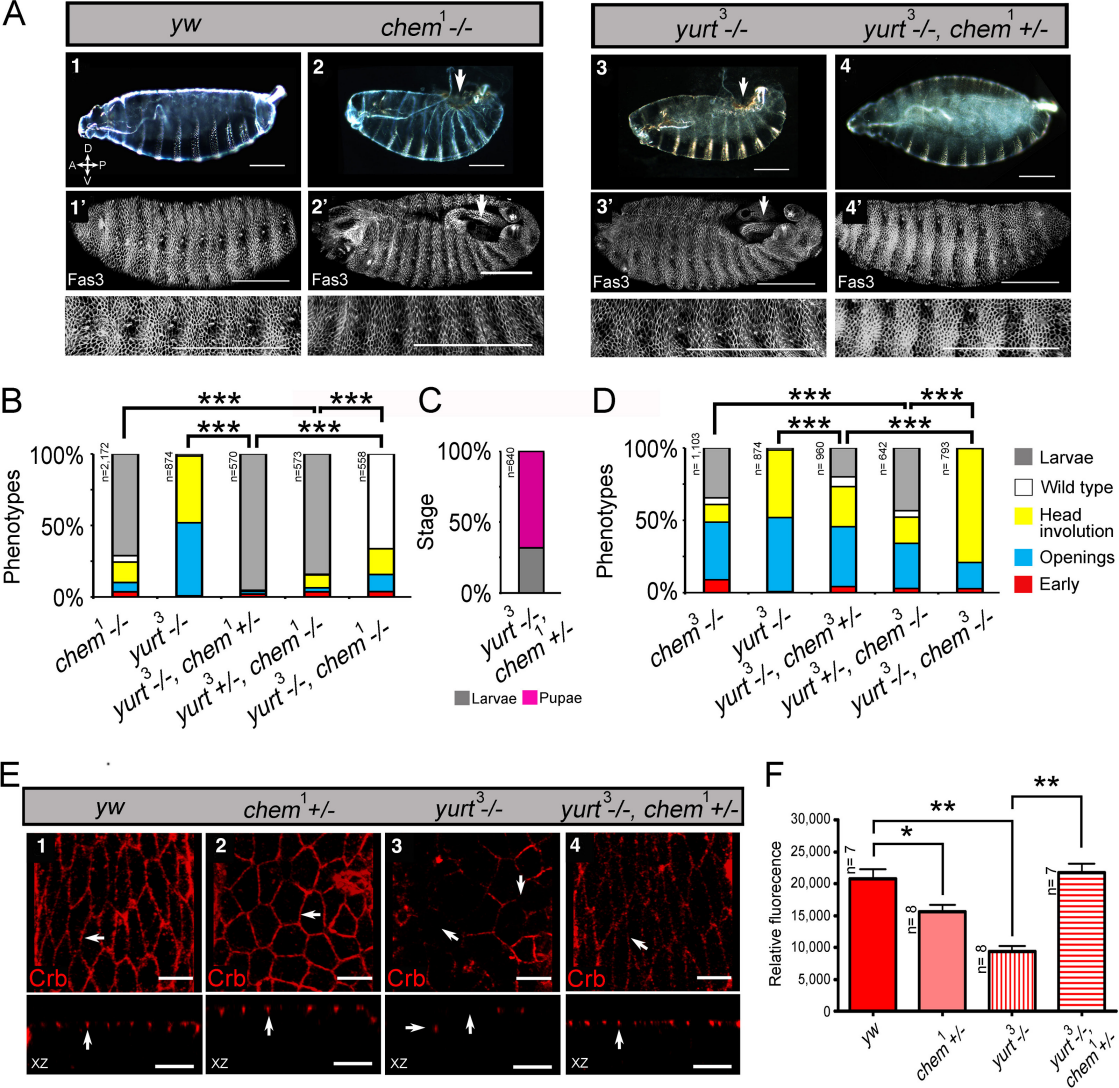
*chem* interacts antagonistically with *yurt*. (A) Control (*y w*; 1), *chem*^1^(2), *yurt^3^* (3) and a *yurt^3^* homozygous mutant, *chem*^1^ heterozygous (4) mutant embryos, stages 15–16, were stained with anti-Fas 3 to evidence the lateral epithelium during dorsal closure stages. The top line shows embryonic cuticles, whereas the second line shows a panoramic dorsal view (anterior to the left) of Fas 3 stained closing (1^′^, 4^′^) or not closing (2^′^, 3^′^) embryos. Bottom row shows a higher magnification of the corresponding lateral epithelia stained with antibodies against Fas 3. Notice similar dorsal closure defects in *chem*^1^ and *yurt^3^* embryos (white arrows; 2, 2^′^ and 3, 3^′^ panels). Representative examples are shown. Scale bars are 100 micrometers. (B) Heterozygosity for *chem*^1^ significantly partially suppresses *yurt^3^* mutant phenotypes. The left graph shows the *chem*^1^and *yurt^3^* embryonic and larval mutant phenotypes, and the suppression of *yurt^3^* mutant phenotypes by heterozygosity for *chem*^1^ in a *yurt^3^* mutant background. (C) About three quarters of suppressed *yurt^3^* homozygotes, heterozygotes for *chem*^1^ first instar larvae, selected and transferred to fresh food vials die as pupae, whereas homozygous *yurt^3^* are all embryonic lethal (B). (D) A similar, significant, but weaker effect is seen with *chem*^3^ heterozygosity in a *yurt^3^* mutant background. In (B) and (D) there is also a significant suppression of *chem* homozygous mutant phenotypes by *yurt^3^* heterozygosity. (E) Staining with antibodies against Crumbs in stages 13–14 lateral epithelial cells show reduction of staining in embryos homozygous mutant for *yurt^3^* and suppression by heterozygosity for *chem*^1^. Control (*y w*; 1), *chem*^1/+^(2), *yurt^3^* (3) and a *yurt^3^* homozygous mutant, *chem*^1^ heterozygous (4) mutant embryos. There is also a significant reduction of Crumbs staining signal in heterozygous *chem*^1^ embryos. White arrows point, in orthogonal views (*Z* axis), to apical Crumbs signal, partially disrupted in embryos homozygous mutant for *yurt^3^*. Representative examples are shown. *n* = 7–8. Scale bar is 5 micrometers. (F) Quantification of the antibody signal of embryo classes as in (E). For the whole figure, n is written to the left of all graphs.

*yurt* mutant larvae have embryonic phenotypes similar to *chem* ( (*l(3R)5G83*) (Jürgens et al., 1984). *yurt* codes for a band 4.1 homolog in flies, a group of proteins known to interact with the actin-spectrin cytoskeleton (Hoover & Bryant, 2002), and to form part of a group of genes promoting basolateral identity in epithelial membranes (Laprise et al., 2009). Particularly, embryos with dorsal holes (due to failure of dorsal closure) in the two loci have dorsal holes positioned towards the posterior of the embryo, and not anteriorly or centrally located, like most dorsal closure mutants described (Rios-Barrera & Riesgo-Escovar, 2013). This is clearly seen in embryos stained for Fas 3 to evidence the lateral epithelium (Fig. 4A). Despite the fact that the percentage of embryonic mutant phenotypes is very different, we studied genetic interactions between the two loci.

We initially studied interactions between *chem*^1^ with *yurt*^3^, as it is a weak allele of the locus, and repeated the experiments with the strong *chem*^3^ allele. We found that heterozygosity for *chem*^1^ significantly suppresses *yurt* mutant phenotypes (Fig. 4B). We selected *yurt*^3^ homozygotes heterozygote for *chem*^1^ larvae and cultured them separately. A majority of these mutant larvae reach pupal stages, similar to *chem*^1^ homozygotes, whereas *yurt*^3^ homozygotes all die as embryos (Fig. 4C), showing a suppression of the *yurt*^3^ phenotype by virtue of heterozygosity for *chem*^1^. A significantly similar but weaker suppression of *yurt*^3^ is seen with heterozygosity for *chem*^3^ (Fig. 4D).

In comparison, heterozygosity for *yurt*^3^ leads to a weak, but significant suppression of *chem* homozygotes embryonic mutant phenotypes (*chem*^3^ and *chem*^1^; Figs. 4B and 4D). The double homozygotes show significant rescue from the *yurt*^3^ mutant phenotype, but are an enhancement of the corresponding *chem* mutant phenotypes.

### *chem* and *crumbs*

In order to study in more detail the *chem* and *yurt*/*chem* epithelial phenotypes, we stained several of these mutant embryos for the transmembrane protein Crumbs. Crumbs is an EGF-repeat rich transmembrane protein found in the marginal zone of epithelia, required for epithelial polarization that interacts with Yurt (Laprise et al., 2006; Tepass, Theres & Knust, 1990). As expected, Crumbs expression is significantly reduced in *yurt*^3^ mutants (Figs. 4E and 4F). A smaller, but significant Crumbs expression reduction is also seen in *chem*^1^ heterozygotes. The reduced Crumbs expression and mislocalization is suppressed to wild type levels in *yurt*^3^ mutants by heterozygosity for *chem*^1^ (Figs. 4E and 4F). Taken together, this is all consistent with *chem* interacting with the epithelial polarity and/or the cytoskeleton during dorsal closure, and acting counter to *yurt* in epithelial polarity.

**Figure 5:**
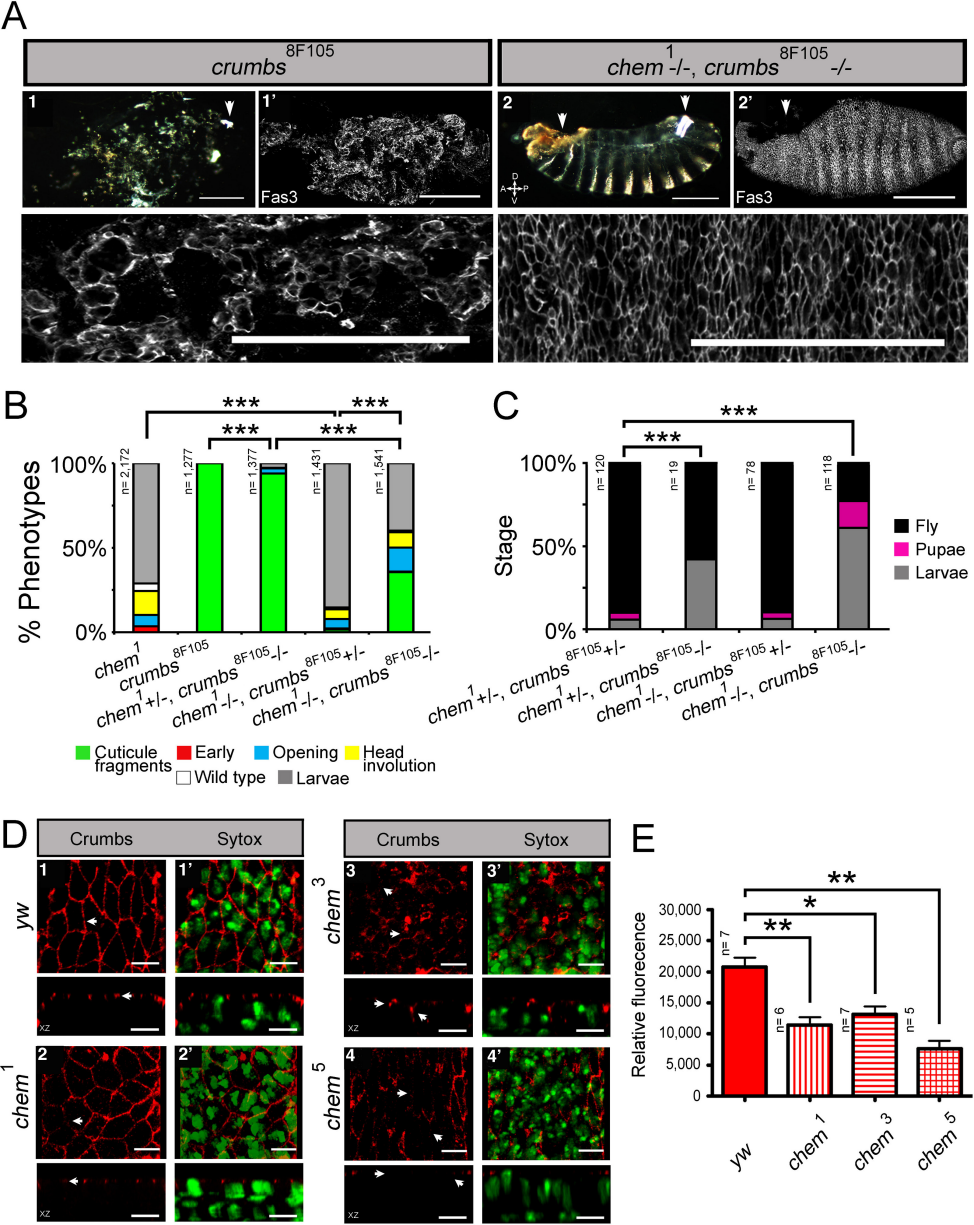
Heterozygosity for *chem*^1^ interacts antagonistically with homozygous *crumbs*^*8F105*^, partially suppressing *crumbs*^*8F105*^ phenotypes and lethality. (A) Shows the *crumbs*^*8F105*^ mutant cuticular phenotype, 1, with white arrowhead pointing to a filzkörper, and 1^′^to a Fas 3 staining of stage 15–16 embryo. Only characteristic small pieces of cuticle (or epithelium) are seen. Lower row shows a higher magnification of the mutant epithelium in 1^′^. Embryos doubly homozygous mutant for *chem*^1^ and *crumbs*^*8F105*^ exhibit suppression of the cuticular *crumbs*^*8F105*^ phenotype, 2. Left arrowhead points to a dorsal anterior hole and right arrowhead to the filzkörper. Staining with antibodies against Fas 3 show suppression of the *crumbs*^*8F105*^ epithelial phenotype in a stage 15–16 embryo, 2^′^. White arrowhead points to dorsal anterior hole. Lower row shows a higher magnification of the epithelium stained with antibodies against Fas 3. Representative examples are shown. Scale bars are 100 micrometers. (B) *chem*^1^ suppresses *crumbs*^*8F105*^embryonic phenotypes, Heterozygosity for *crumbs*^*8F105*^ also suppresses *chem*^1^ phenotypes. (C) Surviving mutant larvae were separated from balancer chromosome siblings, cultured, and studied. Heterozygosity or homozygosity for *chem*^1^ rescues partially *crumbs*^*8F105*^ lethality, compared to *crumbs*^*8F105*^ homozygotes, where all die as embryos. Sibling control larvae doubly heterozygous for *chem*^1^ and *crumbs*^*8F105*^ show higher percentage of survival. Heterozygosity for *crumbs*^*8F105*^ also rescues *chem*^1^ lethality, to levels similar to *chem*^1^ and *crumbs*^*8F105*^ heterozygotes. (D) Crumbs expression is reduced in *chem* mutants, and mislocalized (seen clearly in *chem*^3^ homozygotes) in stages 13–14 embryos. 1–4 show staining with antibodies against Crumbs, top rows XY views, and bottom rows orthogonal XZ views. In 1, white arrows point to Crumbs staining pattern and apical localization, whereas in 2–4 arrows point to Crumbs staining reduction and mislocalization (this last evident in 3). 1^′^–4^′^ show both the Crumbs staining and the Sytox nuclear staining. Representative examples are shown. Scale bar is 5 micrometers. (E) Shows the quantitation of the Crumbs staining from (D) genotypes. In all *chem* homozygote mutant embryos studied, Crumbs staining is significantly reduced.

Next, we studied genetic interactions between *chem* and *crumbs*. We used the hypomorphic *chem*^1^ allele and the *crumbs*^8*F*105^ embryonic lethal allele (Tearle & Nusslein-Volhard, 1987; Tepass, Theres & Knust, 1990). *crumbs*^8*F*105^ homozgote embryos die with small pieces of cuticle formed, seen in a cuticle preparation and by Fas 3 staining (Fig. 5A). This very penetrant phenotype is significantly suppressed by heterozygosity for *chem*^1^, more so in the double mutant homozygote (Figs. 5B and 5B). In fact, a fraction of *crumbs*^8*F*105^ homozygous mutant larvae heterozygous or homozygote for *chem*^1^ cultured in separate tubes without larvae of other genotypes, reaches the adult stage (Fig. 5C). Normally, all *crumbs*^8*F*105^ mutant embryos die before hatching. *crumbs*^8*F*105^ heterozygosity also suppresses partially the homozygous *chem*^1^ mutant phenotype (Fig. 5B), and surviving larvae, cultured separate from other genotypes, reach the adult stage at the same rate as sibling double heterozygotes (Fig. 5C).

We next studied Crumbs expression in *chem* mutants. Homozygous mutant *chem*^1^, *chem*^3^, and *chem*^5^ have basally mislocalized and reduced Crumbs expression (Figs. 5D and 5E). Together, this is consistent with *chem* acting counter to *crumbs*, promoting epithelial basolateral membrane. This is also consistent with *chem* having a more general role in epithelial polarity.

### *chem* and *coracle*

*coracle* (*cora*) codes for another band 4.1 type protein, known to distribute to the septate junctions in epithelia, and to promote epithelial polarization (Lamb et al., 1998; Ward, Lamb & Fehon, 1998; Ward et al., 2001). *cora* works in a different pathway from *yurt* (Laprise et al., 2009). We studied genetic interactions between *cora*^2^, a loss of function allele, with fully penetrant embryonic lethality (Fehon, Dawson & Artavanis-Tsakonas, 1994; Lamb et al., 1998) and *chem*^1^. *cora*^2^ and *chem*^1^ significantly enhance each other, including the double homozygote (Fig. 1A). We also stained epithelial cells mutant for *chem* with anti-Cora antibodies, and studied both Cora localization and epithelial integrity and anatomy in the lateral epithelium during dorsal closure stages.

As seen in Fig. 5D and 1B, significantly, *chem* mutant epithelial cells from a disorganized epithelium. Cora protein is mislocalized more basolateral in cells in *chem* mutants in stages 13–14, particularly in *chem*^3^. This epithelial polarity disruption is consistent with a more basal apical-basolateral septate junctions border region in the epithelia, and consequently, an expanded apical domain and a diminished basolateral domain.

We also quantified levels of Cora staining, as we did for Crumbs. We found a non-significant tendency in *chem*^1^ and *chem*^3^ for higher Cora expression. This tendency becomes significantly different in *chem*^5^ in stages 13–14 (Fig. 1D). Contrary to Crumbs staining, Cora is expressed more in *chem* mutants at this stage, pointing to a more general role of Chem in epithelial polarity, as Cora and Yurt/Crumbs work in separate pathways. Yet, this higher expression level is not borne out later: in stage 16 embryos, where Cora has been relocated to the septate junctions in wild-type (Tiklova et al., 2010), there is significantly less Cora staining in *chem* mutant embryos (Fig. 1C, quantitated in Fig. 1D). Compared to wild type, where signal intensity does not change significantly from stages 13–14 to 16, in all *chem* alleles studied, the staining intensity is significantly lower in stage 16 compared to their respective stages 13–14 (about half). This even becomes significantly lower for *chem*^3^ compared to the *yw* control. This is consistent with Cora not being transported apically to the same extent as wild type during embryonic development.

Consistent with the reduced Cora expression in the septate junction compartment, at stage 16, Cora expression in another columnar epithelium, the salivary glands, is also significantly reduced in *chem*^3^ (Figs. 2A and 2B). Also, the extent of staining seen in optical sections through the middle of the embryonic salivary glands, as compared to the distance between the apical extent of the Cora pattern and the end of the salivary glands nuclei, is also significantly reduced in *chem*^5^ (Fig. 2C). This is consistent with reduced Cora expression in these epithelial cells as well. Taken together, these results support a requirement for Chem in the re-deployment of Cora from the basolateral membrane to the septate junctions, and from an abundant expression at stages 13–14 to a reduced expression at stage 16.

### *chem* and *armadillo*

**Figure 6:**
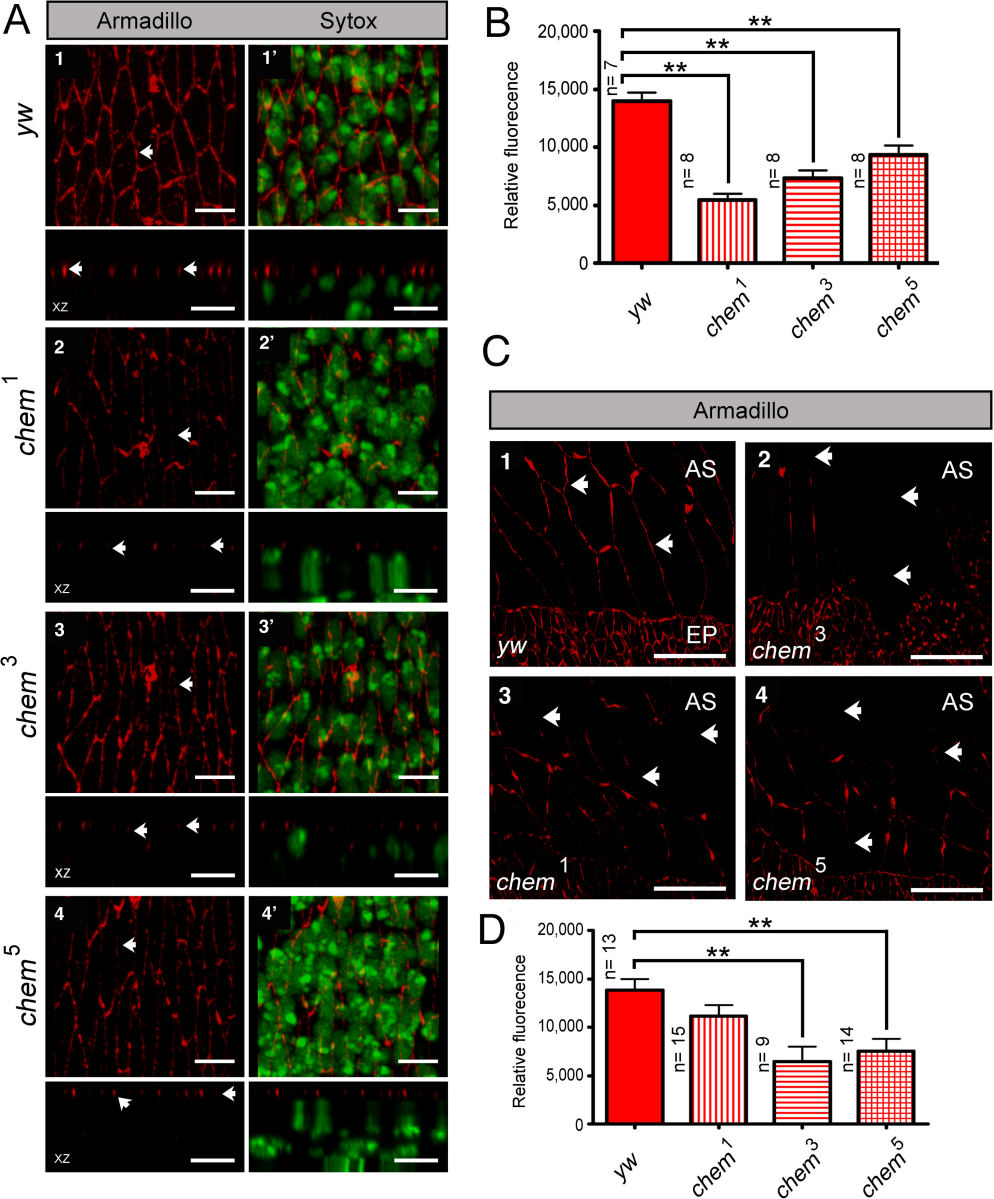
Armadillo (Arm) localization, but not protein levels, is normal in chem mutants. In (A) 1–4 the left top panels are head on views of confocal stacks of stage 13–14 embryos showing staining for Arm, with the corresponding orthogonal views (XZ) underneath. In the left side, 1^′^–4^′^, are the same stacks also showing the same embryos with the Sytox channel included, marking nuclei. Notice disarray of epithelial architecture in *chem* mutants, but the normal apical localization of the Arm staining despite the reduction in staining level. Representative examples are shown. In panels, arrows point to the Arm staining pattern. Scale bar is 5 micrometers. (B) shows quantification of the Arm staining from the experiment in (A). In all cases, Arm staining in *chem* mutant homozygotes is significantly reduced compared to the *y,w* control embryos (*n* = 7–8). (C) 1–4 are head on views of confocal stacks showing Arm staining in amnioserosa cells, with the bottom part showing the dorsalmost part of the lateral epithelium, for orientation. Arrows point to part of the Arm staining pattern (or lack of staining). Representative examples are shown. Scale bar is 20 micrometers. (D) shows the quantification of the Arm staining pattern of the experiment in (C). For the quantification, the study areas were chosen to include only amnioserosa cells and not neighboring lateral epithelium cells. In all cases, staining is reduced, but is significant in *chem*^3^ and *chem*^5^. *n* = 9–15.

We also stained *chem* mutant epithelia with another marker of polarized epithelia, *armadillo* (*arm*). Arm is the *Drosophila β*-catenin homolog expressed subapically in epithelial adherens junctions (Peifer et al., 1991; Peifer & Wieschaus, 1990). The staining pattern and localization of Arm in *chem*^1^, *chem*^3^ and *chem*^5^ is normal, despite the altered epithelia in *chem* mutants (Fig. 6A). Yet the level of Arm staining is significantly reduced in *chem* mutant embryos (Fig. 6A), showing that the expression of this protein is also affected.

The Arm staining level is also disrupted in the amnioserosa. The big, squamous epithelial cells of the amnioserosa, dorsal to the lateral epithelia, had also a reduction of Arm staining, a reduction significant for *chem*^3^ and *chem*^5^ (Fig. 6B). This shows that Chem function is required both in the amnioserosa and the lateral epithelium.

Finally, we measured cell length by an indirect method in the lateral epithelia: we stained lateral epithelial cells with Fas 3, a protein that marks the lateral membranes, and measured “cell length” as the length of Fas 3 staining in Z projections taken from stacks (underlying yolk does not stain with Fas 3) in stages 13–14 of embryogenesis. Using this method, we estimated wild type cell length at 6.1 ± 0.1 micrometers, and *chem*^1^ and *chem*^3^ as slightly longer, *chem*^1^ at 6.5 ± 0.2, and *chem*^3^ at 6.7 ± 0.2. Only *chem*^3^ is significantly different. *chem*^5^ mutant cells are significantly shorter, at 5.5 ± 0.1 micrometers (S1).

## Discussion

*chem* mutants have an expanded phenocritical lethal period, which may be ascribed to disruptions in epithelial polarity, here evidenced by studies in the lateral epithelium during dorsal closure and stage 16 embryos, and by genetic interactions with two epithelial polarity, FERM-domain containing genes: *yurt* and *cora*, and the apical determinant *crumbs*. In spite of these mutant states, some zygotic mutant embryos do survive, and in the strongest allele isolated to date, lead to death in larvae; in other alleles a fraction survives to adulthood. At face value, this suggests that either all the *chem* alleles are hypomorphs and that a true null would be an early lethal, or that the role of *chem* is modulatory.

*chem* germline clones have much stronger phenotypes, consistent with a strong maternal contribution. Maternal contribution masks early requirements for *chem*, allowing a fraction of mutant embryos to survive embryogenesis, and if cultured separately, larval and pupal stages, and to reach the adult stage (adults die within a few days after eclosion). This argues for a clear and important embryonic Chem function during embryogenesis.

The disruptions in epithelia in *chem* mutants suggest a regulatory role for *chem*, on the one hand suppressing *yurt* and *crumbs* phenotypes, and on the other enhancing *cora* phenotypes. Yurt is thought to antagonize apical membrane, in part by its association with the apical determinant Crumbs (Müller, 2003; Tepass, Theres & Knust, 1990). Yurt and Crumbs are components of an epithelial polarity groups of genes different from Cora (Laprise et al., 2009), and whether directly or indirectly, *chem* affects both groups. A plausible explanation is that Chem promotes basolateral membrane in epithelia, as does Cora, located at septate junctions towards the end of embryogenesis. Our observations at stage 13–14 of embryogenesis, as septate junctions are forming (Tiklova et al., 2010), show augmented Cora expression. In contrast, in stage 16 embryos, after the septate junctions are formed and Cora is re-directed to them, in *chem* mutants there is dramatically and significantly less Cora (Figs. 1C and 1D). These observations might provide the beginning of a rationale for the paradoxical finding in this paper that mutations in *chem* enhance *cora* mutations phenotypes, but suppress *yurt* mutant phenotypes. Cora and Yurt are related FERM domain proteins, mutant alleles of which cause similar phenotypes, and are partially redundant, yet *chem* mutants enhance mutations in *cora* but suppress mutations in *yurt*. If Chem regulates Cora re-localization to septate junctions (but not Yurt localization), this Chem requirement for Cora re-localization might explain the enhancement seen in *chem* and *cora* mutant genetic interactions.

**Figure 7:**
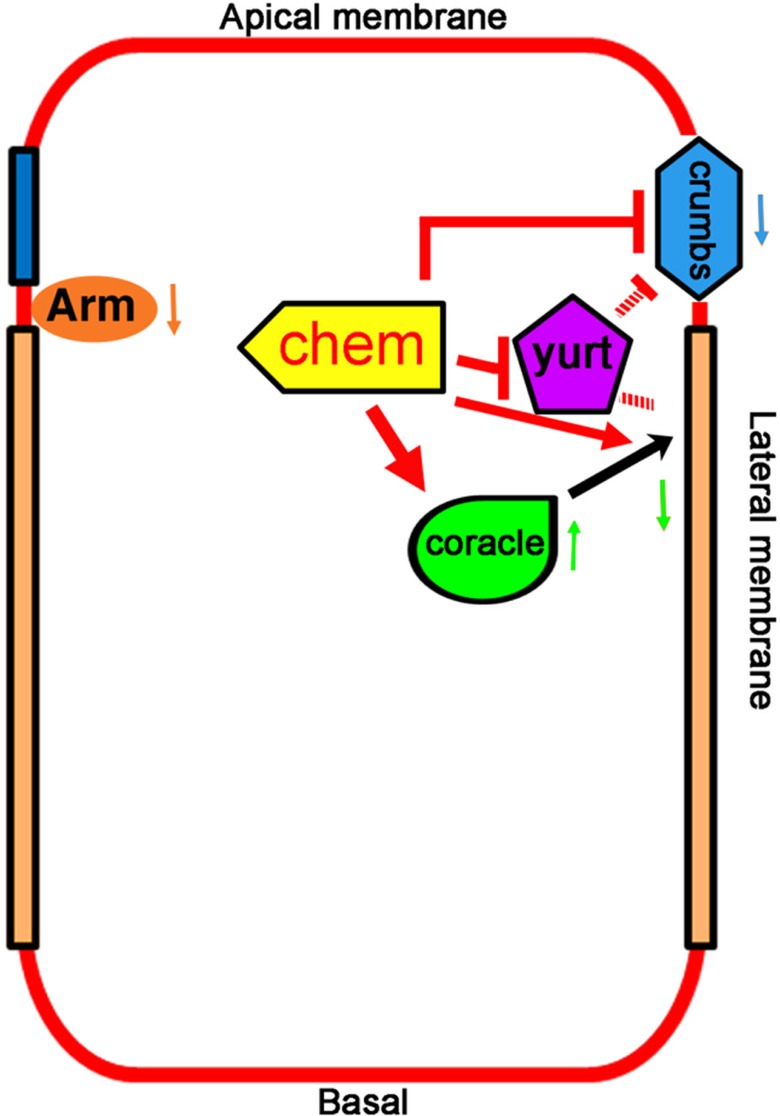
Promotion of basolateral membrane identity by Chem and Cora. A model depicting the genetic interactions studied. A lateral epithelial cell shows the interaction between Cora and Chem, Cora attachment to the septate junctions, and promotion of the basolateral membrane identity by Chem and Cora (orange arrows), with Yurt interactions with Chem and Crumbs, and antagonistic interactions between Chem and Crumbs, and Chem and Yurt. Chem regulates Arm expression levels down (downward pointing orange arrow next to Arm), as it does to Crumbs protein expression (blue arrow pointing downward) and augments Cora expression at stages 13–14 (green arrow pointing upward), then leads to lower Cora expression by stage 16 (green arrow pointing downward). The interactions are genetic, and so are not meant to be direct interactions, protein-protein or otherwise.

Septate junctions are among several of intercellular junctions in epithelia, and although it was suggested originally that among other functions, they serve to compartmentalize domains in epithelial cells (Tepass et al., 2001), subsequent studies have failed to demonstrate such a role (Izumi & Furuse, 2014). Rather, several septate junctions components may have additional roles in establishing and/or maintaining epithelial polarity, as is the case of Coracle (Ward et al., 2001). In embryonic stages 13–14, Cora localization is clearly different in *chem* mutants compared to control embryos, implying another Chem function in relation to Cora (more basal location, and more abundant expression, significantly augmented in *chem*^5^ mutations). In summary, Chem is required for the stages 13–14 localization and level of Cora, and subsequently, for its re-deployment, leading to septate junction Cora localization by stage 16. Chem might interact differently with Yurt, a member of an epithelial polarity gene group different from Cora, and thus, lead to different genetic interaction and results.

Crumbs is an apical marker, and is mislocalized basally in *chem* mutants, consistent with an expanded or disorganized apical domain in *chem* mutants. Levels of Crumbs expression are also lower in *chem* and *yurt* mutants, a phenotype rescued in the *chem* heterozygous suppression of *yurt* homozygous mutants. Particularly noteworthy is the recovery of adult flies homozygous for *crumbs*^8*F*105^, heterozygous for *chem*^1^. Similar to the case of *yurt* and *chem* interactions, *crumbs* and *chem* suppress each other. By combining conditions that weaken the apical domain (mutations in *yurt*) with conditions that weaken basolateral membrane (*chem* (or *cora*) mutant conditions), membrane domains can be re-established, at least partially, and may explain both the *chem* mutations suppression of *yurt*^3^mutation, and the enhancement of *chem* and *cora* mutant phenotypes.

Despite this, adherens junctions appear to be present, as Arm staining has a normal pattern in *chem* mutants, even though Arm levels are reduced. The overall picture suggests that *chem* epithelial cells have partially disrupted polarity, with apical markers located more basally, without clear changes in cell size (one allele has longer cells, another shorter, and a third without significant changes). There is also an imbalance in the epithelial polarity genes examined: reduced expression at stages 13–14 for Crumbs and Arm, but augmented expression of Cora at stages 13–14, then subsequent reduced expression of Cora, leading to the overall consequence that *chem* mutations result in reduction of protein levels of the polarity genes here examined. Clearly, the roles played by Chem in epithelial polarity are complex, and warrants further study.

Finally, the new hypomorphic alleles, with effects that do not necessarily result in death, but rather render the individuals prone to culling from competition with healthier ones, should allow the study of other processes disrupted in *chem* mutants throughout the life cycle. It will be of interest to study how other epithelia are affected, besides the lateral epithelia, salivary glands, and the amnioserosa.

## Conclusions

We have isolated new alleles in the *chem* locus and shown that these mutations disrupt epithelial polarity, a phenotype that may explain its deleterious effects. In *chem* mutants the septate junctions protein Cora and the apical protein Crumbs are mislocalized more basally, and end with a reduced expression, consistent with an altered balance in apical/basolateral membrane domains in epithelia. The Arm expression pattern is normal, but expression levels of Arm are lower. Despite similar mutant phenotypes, *chem* and *yurt* mutations behave antagonistically. *crumbs* and *chem* mutations also behave antagonistically. *chem* and *cora* mutations enhance the mutant phenotypes of each other (Fig. 7).

##  Supplemental Information

10.7717/peerj.2731/supp-1 Data S1 Raw data of intensity fluorescence measurements of coracle staining in salivary glands

10.7717/peerj.2731/supp-2 Data S2 Raw data of fluorescence intensity measurements in embryos

10.7717/peerj.2731/supp-3 Supplemental Information 1 Complementation table of chem alleles

10.7717/peerj.2731/supp-4 Table S1 Complementation of *chem* alleles All six *chem* mutant alleles were crossed between them and tested for lethality complementation. NC, non-complementing, meaning that the heteroallelic combination is lethal (no heteroallelic adults were recovered). In cases where transheterozygote adult escapers were recovered, the percentage of those is annotated. In all cases, and if adult escapers were recovered, the percentage of escapers is significantly under the expected percentage for complementation (33.3%). Number of organisms scored per cross (*n*) was between 60–142.

10.7717/peerj.2731/supp-5 Figure S1 Cell size measurements in *chem* (A) Shows representative head on views of confocal stacks of stages 13–14 embryos of wild type control (*y,w*) and homozygous *chem* mutant alleles stained for Fas 3 and Sytox nuclear stain. **a**–**d** top rows, left side, show the Fas 3 staining, whereas the bottom rows left side show orthogonal views (XZ) of the same stack. The **a^′^**–**d^′^** right sides show the same, but include the Sytox nuclear stain channel. Arrows point to the Fas 3 staining pattern in the panels. Scale bar is 5 micrometers throughout. (B) Quantification of the “cell length” as measured by the length of the apical-basal Fas 3 staining. *chem*^1^ mutant embryos are not significantly different form controls, whereas *chem*^3^ mutant embryos have a significantly longer Fas 3 staining, and *chem*^5^ a significantly shorter Fas 3 staining. Number of replicates (n) is written to the left of the corresponding column.

## Additional Information and Declarations

### Data Availability

The following information was supplied regarding data availability: The raw data has been supplied as Supplemental File.
