# Supplementary material for: Drosophila chem mutations disrupt epithelial polarity in Drosophila embryos
Source: PeerJ. 2016 Dec 1;4:e2731. doi: 10.7717/peerj.2731 (PMC5136136; doi:10.7717/peerj.2731)
Supplement: Supplemental Information 1 [file peerj-04-2731-s003.docx]

Crude data. *chem* c.omplementation

|  | *chem ^1^* | *chem ^2^* | *chem ^3^* | *chem ^4^* | *chem ^5^* | *chem ^6^* |
| --- | --- | --- | --- | --- | --- | --- |
| *chem ^1^* | 0/100 | 0/70 | 0/85 | 0/80 | 0/70 | 0/90 |
| *chem ^2^* | 0/100 | 0/90 | 3/80 | 0/112 | 1/60 | 0/90 |
| *chem ^3^* | 0/100 | 1/60 | 0/100 | 0/133 | 4/105 | 1/82 |
| *chem ^4^* | 0/90 | 1/117 | 1/101 | 0/100 | 1/101 | 1/142 |
| *chem ^5^* | 0/90 | 0/94 | 1/124 | 5/95 | 0/89 | 0/95 |
| *chem ^6^* | 0/100 | 0/110 | 0/106 | 1/137 | 0/100 | 0/100 |
