# Supplementary material for: Drosophila chem mutations disrupt epithelial polarity in Drosophila embryos
Source: PeerJ. 2016 Dec 1;4:e2731. doi: 10.7717/peerj.2731 (PMC5136136; doi:10.7717/peerj.2731)
Supplement: Table S1 — All six chem mutant alleles were crossed between them and tested for lethality complementation. NC, non-complementing, meaning that the heteroallelic combination is lethal (no heteroallelic adults were recovered). In cases where transheterozygote adult escapers were recovered, the percentage of those is annotated. In all cases, and if adult escapers were recovered, the percentage of escapers is significantly under the expected percentage for complementation (33.3%). Number of organisms scored per cross (n) was between 60–142. [file peerj-04-2731-s004.docx]

|  | *chem ^1^* | *chem ^2^* | *chem ^3^* | *chem ^4^* | *chem ^5^* | *chem ^6^* |
| --- | --- | --- | --- | --- | --- | --- |
| *chem ^1^* | NC | NC | NC | NC | NC | NC |
| *chem ^2^* |  | NC | 3.75% | 0.8% | 1.6% | NC |
| *chem ^3^* |  |  | NC | 0.9% | 3.8% | 1.2% |
| *chem ^4^* |  |  |  | NC | 5.2% | 0.7% |
| *chem ^5^* |  |  |  |  | NC | NC |
| *chem ^6^* |  |  |  |  |  | NC |

NC: No complementation.

%: of adults emerging from the cross.
